# The Nursing Supplement

**Published:** 1888-07-28

**Authors:** 


### The Nursing Mirror.

All Contributions for this Supplement should be addressed "The Nursing Mirror," care of The Editor, 38, Old Jewry, E.C.

### En Passant.

THE MIDDLESEX INSTITUTE.—Ever since this institute was opened last Michaelmas, it has been under the care of Miss Steuart, who has, however, now resigned her post of sister-superintendent, in order to join Miss Pollock's staff of nurses. The nurses belonging to the Middlesex Hospital Trained Nurses' Institute number about twenty-four; and, besides their fixed salary, they receive a percentage on what they earn. The home is beautifully decorated, and conveniently arranged, but the institute has proved so popular that there are seldom many nurses found enjoying these comforts.

CURED BY THERMOMETER.—The importance attached to a clinical thermometer by those in ignorance of its office approaches a superstition. They close their lips tightly upon it. Their eyes roll wildly around the room. They believe that the tube contains some mighty gas or a metal of mysterious power. "There ain't much taste to it, docther," said one of the credulous fellows, "but I s'pose it's *turrible sthrong*." Dr. ——, who is something of a wag, encouraged the man's faith in the occult virtues of the thing, and with remarkable results. After the first "dose," the fever abated. The "treatment" was continued, and the patient actually recovered, cured by thermometer, administered *ter in die* without further drugging.—From *Scribner's Magazine.*

ENCOUNTER WITH A MADMAN.—As one of the night attendants of the Stone Asylum was going her rounds last week, she found a former patient crouching in a corner of the padded room with a razor in his hand. She screamed and ran, and he followed her into a room full of lunatics, where he knocked her down, and tried to get at her throat with the razor, but could not, owing to a shawl which the night attendant was wearing; and, another assistant coming up, he was pulled away. Dr. Greenlees then came into the room and found the accused sitting on a chest with the open razor in his hand. The man expressed his disappointment at not finding nurse Bragg, whom he said he would kill for reporting his misconduct when he was an inmate of the institution. Whilst conversing with the doctor the prisoner put the razor into his pocket, and was then secured by the attendants. The accused, who is evidently a lunatic, having been confined successively in several asylums, will be sent back to his old quarters.

FLORENCE NIGHTINGALE'S WORKS.—It may interest our readers to have the following full list of the writings of Miss Nightingale: "Notes on Nursing," published by Harrison, 59, Pall Mall, demy octavo, limp cloth, 1860, 2*s.*; cheap edition, revised, 1868, for the use of the labouring classes, with an additional chapter on "Minding Baby," price 6*d.*, in limp cloth 7*d.* "Notes on Hospitals," with appendix on different systems of hospital nursing; Longmans, third edition 1863, post 4to, with 13 plans, 18*s.* (out of print). "Notes on Lying-in Institutions," with a proposal for organising an institution for training midwives and midwifery nurses; Longmans, 1871, 8vo, price 7*s.* 6d., with plans. "The Organisation of Nursing in a Large Town," with an introduction and notes by Florence Nightingale, 1865, Liverpool, Holden, price 2*s.* 6*d.* "Una and the Lion," a paper in "Good Words" for June, 1868. "Training of Nurses," and "Nursing the Sick," articles in Quain's "Dictionary of Medicine," Longmans, 1882.

NURSING IN GERMANY.—Nurses in Germany are almost as much hurt that one of their English sisters was chosen to wait on the late Emperor, as are the doctors with Sir Morell Mackenzie. The best of our nursing systems is merely a copy of the famous "Institute of Deaconesses," founded by Pastor Theodor Fliedner, at Kaiserswerth, in 1836; for it was there that Miss Nightingale completed her early instruction, and gained the experience on which she modelled her system. There are upwards of three thousand deaconesses connected with these schools now, and they make most faithful nurses. There are also many sisters of charity in Germany who undertake nursing, but the most prominent secular school is found in Berlin, at the "Kaiserin Augusta Hospital," where lectures on nursing are combined with practical instruction. Many ladies attend this school and attain a proficiency in nursing not to be excelled in this country, and it was from their ranks that all thought a nurse should have been chosen to wait on the late Emperor, for whom they all felt such sympathy.

A SCHOOL OF MIDWIFERY.—In answer to many inquiries, we wish to state that the Clapham School of Midwifery, which sometimes advertises a vacancy in our pages, is carried on by Dr. Annie McCall, and practice is obtained by attendance on patients at their own homes. For those who are going to India or elsewhere, the three months' course here is excellent, and a student of average intelligence can always pass the London Obstetrical Society's examination at the close of the three months. The students reside at 131, Clapham Road under their lady doctor, and receive class instruction during the intervals when they are not out on their rounds. The school used to be connected with Mrs. Meredith's well-known medical mission, which does such good work in all that neighbourhood. There is a small dispensary not far from the home, and here poor women come to be prescribed for by one of their own sex, and here, too, they prefer to bring their little children. A great deal of useful knowledge can be picked up while resident at the school, over and beyond the thorough training in midwifery which it is its object to give.

THE MAGAZINES AND NURSING.—There can be no doubt that the subject of nursing is much more before the public now than at any past time. It is scarcely possible to take up a magazine which does not contain some paper or story connected with nurses or nursing; and most of these papers are pretty truthful in their particulars. The *Sunday Magazine* for July had an article called "A Lady's Hospital," giving an account of Christmas at the New Hospital for Women; the *Westminster Review* had an article entitled "Nurses and Nursing"; and in the *Sunday at Home* Mrs. Brewer's papers are continued. Mrs. Brewer has not yet reached that part of her subject we are so anxiously waiting for, namely, hospital nurses on the Continent. This month's paper is taken up with particulars concerning St. Bartholomew's, St. Thomas's, the London, the Middlesex, and King's College training schools. It is a very good paper for a would-be probationer to read before deciding on which hospital to apply to, but it contains little that is new to nurses. In last week's *Illustrated London News* the middle sheet was occupied by a series of sketches drawn in the wards of the London Hospital. The sketches of a sister and a probationer both represented rather severe types, not the smiling beauty which is usually supposed to attract a sentimental public.

### Original Articles.

### NURSES IN NOVELS.

THEY are pretty numerous, these nurses in novels, but they have one trait common to the whole community—they are none of them trained, they are all born nurses. This is, of course, to be expected in works of fiction, but still it is rather trying to the real article to find her life's aim and labour so lightly passed over, and represented by a few sentimental touches. There is Catherine in the much-read "Robert Elsmere," who is described in the opening chapters as the Sister Dora of that countryside; being to her poorer neighbours just what Sister Dora was to the thousands of Walsall. But Catherine has never been trained at a hospital, but apparently led a quiet rural life, far from ambulance classes even; and whatever may have been Sister Dora's faults, she at least underwent a long and severe course of training before she began to set fractured limbs and deride doctors' opinions. It is evident that the girl of the period has been to school with Mr. Besant at the People's Palace, and unless she is philanthropical no one will read about her. We no longer take any interest in wicked Becky Sharps, or gentle Amelia Sedleys; the heroines of to-day must be imbued with the gospel of work, they must be devoted to practical labour for the good of humanity, and since they are creatures of fiction they always perform their great and noble actions without any previous training; at one bound they scale the highest height of heroism, and save endless lives without ever having learnt how. In one of Miss Braddon's novels called "An Open Verdict," the hero, who is a curate, overworks himself and is taken ill with "the fever." In the midst of his delirium two nurses turn up in the garb common to French sisterhoods, and drag him back from the gates of death. Of course one of these nurses is the heroine and lady-love of the curate, and the other is her companion. These two women, ignorant, so far as we are told, of the simplest rudiments of nursing, and without any former experience, in putting on the black veils and white caps of nuns, acquire the knowledge which it usually takes years to learn. The strange thing is that these nurses in fiction always take in the doctors; for instance, here is the comment of the doctor on the two women in question: "Whatever institution they belong to it is an admirable one. I don't think we should have pulled you through if it hadn't been for that excellent nursing. No, upon my word I believe you owe those two women your life." Then there is Sabina Zembra, who certainly goes to a hospital for six months, which is a great concession towards reality on the part of fiction. But the use she makes of the knowledge gained in those six months is this: her dog runs in front of a bicycle, and machine and rider come to the ground together, of course Sabina picks up the young man and nurses him, and finally marries him. That is all that nursing is good for in novels, to further the love story which forms the main action, and to prove without dispute what a noble creature the heroine is. There is one book called "The Story of an African Farm," which is perhaps the most powerful piece of fiction published during this century. It is written by a woman, and, like many works of genius, is full of glaring faults which many a child might have avoided. It has an incident improbable even beyond the limits of fiction, for in her last illness the heroine is nursed by a former lover dressed in women's clothes. "The doctor said of Gregory, after four days, 'She is the most experienced nurse I ever came in contact with.' Gregory, standing in the passage, heard it, and laughed in his heart. What need had he of experience? Experience teaches us in a millennium what passion teaches us in an hour." A grain of truth in an ocean of fiction; suffering may rouse our sympathies to have clearer insight of the pains of others, but without experience sympathy is of little use. If only the pictures drawn in novels were true to life we might skip the indefinable details of ward work, and become nurses by the mere adoption of the name and a black veil. But we should not remain nurses long, for being heroines we should shortly marry, and live happily ever afterwards. Only those who have themselves deliberately chosen nursing as a live-long profession can understand all the absurdity of these modern nurses in novels.

### Hints for the Sick-Room.

By M. M. BASIL, M.A., M.B., C.M., Author of "The Commoner Accidents to Life and Limb."

### ADMINISTRATION OF MEDICINES.

THE internal administration ot remedies demands careful attention. Every medicine coming under your charge for the first time should be carefully examined. Any strong taste, smell, or suspicious appearance, not accounted for by the prescription, should make you doubly watchful of the effect of the earlier doses. Most of you will go through all your work without finding that your vigilance on this point has been of the slightest use, but possibly it may be the ill fortune of one of you to detect an important oversight. Some of the mistakes, comical and serious, that have occurred at times, have arisen from confounding guarana for guaha, the perchloride of mercury (corrosive sublimate) with the subchloride (calomel), strychnine for santonine; coarse liquid carbolic acid has also been mistaken for porter, but not dispensed as such. You must make yourselves familiar with the smell, taste, and striking effects of opium, belladonna, arsenic, strychnine, mercury, and some other medicines which are frequently used in every-day practice.

Invariably read the label on the bottle before you give a dose to the patient, not on the first occasion alone, but on each repetition through the whole course of its administration. If two or more bottles are in use for the same patient, a fatal mistake may occur from non-observance of this caution, as for example, by administering a liniment in place of a mixture. If two or more patients are under your charge at the same time, a serious interchange of bottles may occur accidentally, or by design. Labels which are illegible from any cause should be renewed at once.

Never measure out any medicine in doubtful light. You cannot see the marks on the measure glass in such circum stances; and, what is more serious, you may mistake one bottle for another, or commit the grave blunder of administering by the mouth what is meant for external application only. Many institutions adopt the wise plan of dispensing external remedies in coloured bottles only; fluted bottles are still better, as the feeling of the uneven surface will always guide one even in the dark.

Hold the measure glass perfectly straight in your hand while pouring out a dose. Keep the labelled side of the bottle upwards, or rather, pour the dose out with the bottle held sideways. Never trust to the eye alone for judging of the proper dose of a mixture. There must be no "pouring out the medicine and judging according." A drop or minim, tea spoonful, dessert-spoonful, table-spoonful, and wine-glassful, each represents a definite measure of volume. To get the exact quantity required, you must use a measure of volume, and be guided by principles as rigid as the rules of arithmetic. You must time yourself by the clock in administering medicines.

The bottle must be kept carefully corked, otherwise important alterations in the composition of the mixture may take place through evaporation and other processes. As a general rule, you should shake the bottle before pouring out a dose; in certain cases, on the other hand, you must carefully avoid any rough movement of its contents. The measure glass should be rinsed out and wiped with a special duster each time it is used. A separate measure glass must be kept for oily liquids—cod liver oil, castor oil—or strongly-smelling ones, such as assafœtida and valerian.

(*To be continued.*)

### The Nurses' Bookshelf.

[Books for Review should be sent to The Editor, The Lodge, Porchester Square, W.]

### * Nurses and Nursing.

ONE of the most interesting articles in the *Westminster Review* for this month is the thoughtful paper on "Nurses and Nursing," written evidently by one who knows nurses, their duties, and their wants. The various duties of district, hospital, and private nurses are told in very few words, but with a clear and full conciseness which will open the eyes of those who insist on thinking, in spite of all reason that a nurse's chief occupation is to look as pretty as a picturesque cap and dainty apron will make her. The article also points out the impossibility of nurses saving for their old age out of their present small salaries. "It is estimated," says the writer, "that the average length of a nurse's life in a hospital is about twelve years, and during that time her services are remunerated at about the rate of £25 per annum. This is where the evil of the hospital system comes in: it uses up the best years of a woman's life, and then leaves her to secure support in her old age as best she can." He then draws attention to the National Pension Fund, showing how necessary and valuable such a fund is, and alluding to the readiness nurses have shown to take advantage of it. The article concludes with a fine testimony to the worth of nurses, which should make the hearts of all who belong to the profession glow. "It is the nurse whose purity and truth breaks through the crust of scepticism with which our souls were crusted over, and opens once more the streams of love and faith which flooded our being in our youth. Before the keen light of earnestness in a nurse's eyes the froth and frivolity of social life dries up to nothing, and in its stead grow noble aspirations. The influence exercised far and wide by a nurse is almost unbounded, and if she be actuated by the fervent love of humanity which urges many women to undertake this work, she can carry everywhere with her a glorious torch to light all upwards towards more sublime and unselfish aims, lifting them for awhile above the trivial commonplaceness of which too many of us are liable to allow our lives to consist."

### Everybody's Opinion.

[*Correspondence on all subjects is invited, but correspondents must send name and address, and write on one side of the paper only.*]

### SAINT WILLIAM'S HOSPITAL.

ALLOW me to correct your mistake in calling our hospital "*Lieutenant* William's Hospital." The real name is "St. William's." There are the remains of a chapel dedicated to this holy person near the spot where the hospital is built. Moreover the saint was very clever in curing the plague and other archaic diseases. These two circumstances, I suppose, led the authorities to dedicate the hospital to him. It is a new building, just beyond Dulce Farm, at the top of Dulce Lane, below St. Peter's Parsonage. The saint was a godly baker from Perth, who was on his way to worship and lay large alms at the shrine of St. Thomas à Becket in Canterbury Cathedral. On his way thither he was hospitably entreated by the prior and monks of Rochester. But just after he left this city he was cruelly murdered by his serving man, for the sake of the gold which he was about to bestow upon Becket's shrine. Why the serving man murdered him at Rochester deponent sayeth not. It is one of those details of mediæval story which we must take on trust. The envious said that Rochester was jealous of Canterbury, and of the huge sums of money which Becket drew from faithful pockets, and that it wanted an attraction of its own. At any rate St. William (of Perth and Rochester) became a very famous saint, and an incredulous nineteenth century blesses the hospital named after him, even if it does not venerate the saint himself.

Rochester. A. CANON.

### GEORGE ELIOT'S THOUGHTS ON NURSING.

THE following paragraph by George Eliot, will I am sure prove interesting to my fellow nurses, so I copy it out and send it to you:—"No wonder the sick-room and the lazaretto have so often been a refuge from the tossings of intellectual doubt, a place of repose for the worn and wounded spirit. Here is a duty about which all creeds and all philosophies are at one: here, at least, the conscience will not be dogged by doubt, the benign impulse will not be checked by adverse theory: here you may begin to act without settling one preliminary question. To moisten the sufferer's parched lips through the long night watches, to bear up the drooping head, to lift the helpless limbs, to divine the want that can find no utterance beyond the feeble motion of the hand, or beseeching glance of the eye, these are the offices that demand no self-questioning, no casuistry, no assent to proposition, no weighing of consequences. Within the four walls where the stir and glare of the world are shut out, and every voice is subdued, where a human being lies prostrate, thrown on the tender mercies of his fellow, the moral relation of man to man is reduced to its utmost clearness and simplicity; bigotry cannot confuse it, theory cannot pervert it, passion awed into quiescence can neither pollute nor perturb it. As we bend over the sick-bed, all the forces of nature rush towards the channels of pity, of patience, and of love, and sweep down the miserable choking drift of our quarrels, our debates, our would-be wisdom, and our clamorous selfish desires. This blessing of serene freedom from the importunities of opinion lies in all simple direct acts of mercy, and is one source of that sweet calm which is often felt by the watcher in the sick-room, even when the duties there are of a hard and terrible kind."—*Janet's Repentance.* NURSE LEE.

### Notes and Queries.

TO CORRESPONDENTS.—1. Questions may be written on post-cards. 2. Advertisements in disguise are inadmissible. 3. In answering a query please quote the number. 4. If a private answer is desired, a stamped addressed envelope must be forwarded.

(37) *Massage.*—Is there any hospital where a child could receive massage treatment free, or for a small payment, yet need not be taken as an in-patient?—*J. F. C.*

(38) *Books for Probationers.*—Would some of your readers tell me which they consider the best book for probationers?—*A. G.*

(39) *Private Nursing in New York.*—A nurse wishes to know if there are any institutions for private nursing in New York; also, if the Editor will oblige her with the address of a few of the best.

### Answers.

(34) *Undenominational Sisterhood.*—I should advise *A. H.* to apply to the Charity Organisation Society, or the Kyrle Society. I believe members of both sometimes live together for companionship.

(37) *Massage.*—Apply to the Hospital for Sick Children, Great Ormond Street. They will help you if possible.

(40) *To Correspondents.*—*M. F. S.* and others are warned that letters cannot be inserted unless name and address of writer are sent, not necessarily for publication.

### Exchange and Mart.

UNDER this heading we propose to insert purely Exchange advertisements, at a charge of sixpence for eighteen words, or three insertions for one shilling It must be distinctly understood that ordinary advertisements will not be inserted here.

WANTED.—"Quain's Dictionary," in fair condition. Would give a pocket spectroscope, which cost twenty-five shillings. Address Student, Office of THE HOSPITAL.

APRONS.—Half a-dozen white linen aprons with bibs. Will exchange for nurse's surgical case. Nurse Norma, Office of THE HOSPITAL, 38, Old Jewry, E.C.

* "The Westminster Review" for July, 1888. London: Trübner and Co., Ludgate Hill.

### Appointments.

[It is requested that successful candidates will send a copy of their applications and testimonials, with date of election, to THE EDITOR, The Lodge, Porchester Square, W.]

MISS A. JENNINGS has been appointed matron of Lady Strangford's Hospital, Port Said. Miss Jennings was trained at the Women's Hospital, Cork, and is now in charge of the children's ward of the County Hospital, Maidstone. Miss Jennings is only 24 years of age, and must possess considerable courage to accept such a distant appointment and the charge of some two dozen patients and a staff of nurses and officials. We hope her courage may be rewarded, and all difficulties succumb before her.

### Lectures.

THE lecture at the Midwives' Institute on July 20th was very well attended, the subject, "The Preparation For and After Treatment of Operations in Private Houses," being one on which many hospital nurses must confess themselves ignorant. Mr. Bruce Clarke began by stating that a great deal is expected of a nurse in a private house which is never demanded of her in a hospital. Very often it is the mistress of the house who is ill, all is at sixes and sevens, and the nurse is supposed not only to be capable of nursing, but to be competent to superintend the house as well. It is always better if a nurse can be in a house a day or two before the operation is to be performed, so that she may arrange the various details of the room, and learn her way about. A lady's room is often strewn with nicknacks which cause dust to congregate and are better out of the way. With a little tact, a nurse can generally remove superfluous ornaments and furniture, and clear away clothes or any superfluous hangings which fill up the room. She will see that the bed is of a suitable size and height, not a great clumsy four-poster, and that there is a fire-place, or other means of ventilation. Unfortunately it is not rare to have ventilation reversed; to have inlet shafts becoming outlet shafts, and *vice versâ*. Care, too, should be taken that all the foul air of the house does not ventilate into the bed room. To be quite sure that the air is flowing in at the window and out by the chimney, it is only necessary to hold a piece of smouldering brown paper between the two, and watch the smoke. Why doctors are so particular about the choice and arrangement of a sick room, is because the patient must spend all the time in that room, and unless a large amount of fresh air is let in, the patient must suffer. People who are well covered up in bed seldom suffer from too much air. With regard to food, the nurse should draw up a list for the doctor to sign, so that should persuasion fail she can use authority. A simple pudding, much appreciated by patients, consists of half a pound of bread, one ounce of butter, two ounces of sugar, three eggs, and a pint of milk. The milk should be poured hot over the butter and bread, the sugar and eggs may then be added, and the whole thoroughly steamed. Serve hot with sauce flavoured to taste. A nurse can also save a patient from injudicious friends, who are liable to cause a slow convalescence. We all have likes and dislikes, and when we are ill we like to be humoured. The following is a list of things a nurse should either have at hand, or know where to procure if wanted at a moment's notice:—scissors, forceps, enema and morphia syringes, brandy, ice, hot and cold water, medicine glass, india-rubber water-bottle, bath, water-bed, air cushion, bed-pan, and sofa for a convalescent patient.

### Vacancies.

The following vacancies are announced:—

**Nurse-Housekeeper.**

Teddington Cottage Hospital; £18.

**Matron.**
Halstead Cottage Hospital; £25.

**Nurses.**

Bedford Infirmary; £22.

Bournemouth Sanatorium.

Nurses' Home, Sheffield.

Newcastle Infectious Hospital.

Ipswich Nurses' Home; £22.

Swansea Nursing Institute.

St. Helier's Nursing Institute.

Brighton and Hove Lying-In Institution; £30.

Edinburgh Poorhouse; £25.

Canterbury Nurses Institute.

Ipswich Hospital; £25.

Nurses' Home, Truro.

Eltham Cottage Hospital.

Newport Infirmary.

Ryde Nursing Institute.

Kingston Home, Hull.

Clapham, Brixton, and Surrey Association; Several.

Royal Albert Hospital, Devonport; £25.

Victoria Home for Nurses, Bournemouth; Several.

Female Hospital, Woolwich.

**Probationers.**

Ipswich Hospital.
Isle of Man General Hospital.
Canterbury Nurses' Institute.
Grantham Hospital.

**Nurse Superintendent.**
Bradford Infirmary; £60.

### Examination Questions.

[Copies of similar questions will be gladly received by the EDITOR.]

(100 marks allowed for each question.)

The following are questions given to probationers at Marylebone Infirmary:

1. Describe the composition of bone, giving the preparation of organic and inorganic materail in adult bone.
2. What are the functions of saliva and bile; what are the organs ot excretion?
3. What is the meaning of the following terms: Cat Lini, Ry Tincturæ ʒ t. d, s, Emp Lyttæ, Chloral, ℥j., ʒi., opus sit, Guttæ Eserinii, Hæmoptysis, Melæna, Anorexia, Dyspnæa, ℥j., ʒss., O/., gr. xx.?
4. What are the varieties of Bandages, and the uses of each? What are the rules for applying a roller Bandage.

What are the varieties of the Enema? What are the precautions to be taken when giving one?

6. What would you do to a patient who had taken an over dose of opium—carbolic oil, or nitric acid?
7. What are the signs of bed sores, and what precautions should a nurse take to prevent them?
8. Mention the main groups of food and examples of each. Why are milk and eggs so often used as food for invalids? Why is tripe and unskimmed milk given to invalids?

### THE NATIONAL PENSION FUND FOR NURSES.

**PATRONESSES.**

THE DUCHESS OF BEAUFORT. | LADY ROTHSCHILD.
THE COUNTESS OF ROSEBERY. | THE COUNTESS OF STRAFFORD.

**VICE-PRESIDENTS.**

THE EARL OF ABERDEEN. | SIR EDMUND HAY CURRIE. | E. A. HAMBRO, Esq.
LORD ROTHSCHILD. | HENRY HUCKS GIBBS, Esq. | JUNIUS S. MORGAN, Esq.

WALTER H. BURNS, Esq. (Messrs. J. S. Morgan and Co.), *Chairman.*
HENRY C. BURDETT, Esq. (The Founder), *Deputy Chairman.*

### SPECIAL NOTICE TO NURSES.

IT does not appear to be generally understood that all nurses under fifty years of age, who wish to participate in the benefits of the Pension Fund, are invited to join the Fund by paying into it one-eighth of their present earnings per annum. All nurses who really desire to be helped to the utmost to help themselves, and whose circumstances are such as to prevent them from putting by a larger sum than one-eighth of their income, are invited to apply to the Secretary, 38, Old Jewry, E.C., for a special form of application, which should be filled up and returned with as little delay as possible. Those nurses who have the foresight to adopt this course may rest assured that their applications will be most carefully considered, with the view of securing them the utmost possible provision when overtaken by permanent illness or old age.

PHILIP GROVE, *Secretary*, 38, Old Jewry, E.C.

